# Genetic Linkage Analysis of DFNB4, DFNB28, DFNB93 Loci in Autosomal Recessive Non-syndromic Hearing Loss: Evidence for Digenic Inheritance in *GJB2* and *GJB3* Mutations

**Published:** 2018-01

**Authors:** Marzieh NASERI, Masoud AKBARZADEHLALEH, Marjan MASOUDI, Najmeh AHANGARI, Ali Akbar POURSADEGH ZONOUZI, Ahmad POURSADEGH ZONOUZI, Leila SHAMS, Azim NEJATIZADEH

**Affiliations:** 1. Molecular Medicine Research Center, Hormozgan University of Medical Sciences, Bandar Abbas, Iran; 2. Biotechnology Research Center, Tabriz University of Medical Sciences, Tabriz, Iran

**Keywords:** ARNSHL, GJB2, GJB3, DFNB loci, Linkage analysis, Iran

## Abstract

**Background:**

Autosomal recessive non-syndromic hearing loss (ARNSHL) a most frequent hereditary type of hearing impairment, exhibit tremendous genetic heterogeneity. We aimed to determine the contribution of three common DFNB loci (DFNB4, DFNB28, and DFNB93), and mutation analysis of Gap Junction Beta-2 gene (GJB2) and GJB3 genes in ARNSHL subjects in southern Iran.

**Methods:**

Thirty-six large ARNSHL pedigrees (167 individuals) with at least two affected subjects (72 patients) were included in this descriptive study from Hormozgan Province of Iran, during 2014 – 2015. The variation of GJB2 and GJB3 genes were screened using direct sequencing method. The negative samples for GJB2 and GJB3 genes mutations were analyzed for the linkage to DFNB4, DFNB28, and DFNB93 loci by genotyping the corresponding short tandem repeat (STR) markers using polymerase chain reaction (PCR) and polyacrylamide gel electrophoresis (PAGE) methods.

**Results:**

DNA sequencing of GJB2 were identified heterozygous mutation (964 C/T) in 13.88% of the studied families. Three missense mutations (788G/A, 284C/T and 973G/C) were also detected in coding region of the GJB3 gene. The 284C/T mutation in the GJB3 occurs in compound heterozygosity along with the 964T/C mutation in the GJB2 in one family. Finally, we found no evidence of linkage to either of DFNB4, DFNB93 and DFNB28 loci.

**Conclusion:**

Highlighting the hypothesis that a genetic interaction between GJB2 and GJB3 genes could be lead to ARNSHL, however, no evidence of linkage to the DFNB loci was found. 284C/T variant in GJB3 gene might be pathogenic when accompanied by variant in GJB2 in a digenic pattern. However, further large-scale familial and functional studies are required to challenge this hypothesis.

## Introduction

Hearing loss (HL), with an incidence of 1–2 cases in 1000 live births, is the prevalent sensorineural disorder that environmental risk factors, hereditary genetic defect, and gene-environment interaction are implicated in the etiology of it (1). Despite high genetic heterogeneity and multiple phenocopies of HL, the attempt to find candidate loci for HL resulted in discovery of approximately 300 loci (2).

Autosomal recessive non-syndromic hearing loss (ARNSHL) is the most common hereditary type of hearing impairment and more than 700 different causative mutations have been identified in over 80 loci known as DFNB (3). DFNB1 harbors the first most common ARNSHL causing gene, gap junction beta-2 gene (GJB2), which encodes connexin 26 proteins (MIM 121011). Approximately 50% of ARNSHL has been linked to mutations in the GJB2 in many population and over 100 culprit mutations have been reported (http://davinci.crg.es/deafness) (4, 5). As ethnicity is concerned, the contribution of GJB2 gene mutations in pathogenesis of ARNSHL ranges from 16.7% to 18.29% in Iranian cases and 4.17% of total ARNSHL patients carry a single heterozygous mutation of this gene (6–8). Therefore, the GJB2 gene mutations only explain a fraction of ARNSHL and the reported rates highlights the possible role of other loci in this population.

Other loci are associated with ARNSHL including DFNA2B (GJB3, MIM603324), DFNB4 (SLC26A4, MIM605646), DFNB28 (TRIOBP, MIM609761), and DFNB93 (CABP2, MIM607314) (9–12).

The GJB3gene encodes for connexin 31 and mapped on chromosome 1p34. Despite widespread studies to unveil functional impact of GJB3 mutations, its pathogenic role remains elusive (2, 13). Solute Carrier Family 26 Member 4 (SLC26A4) gene is located at 7q22-q31encoding a chloride/iodide transporter protein pendrin that plays a key role in maintaining the endocochlear potential (14, 15). To date, more than 200 mutations have been identified in SLC26A4 gene associated with ARNSHL. Mutations in SLC26A4 gene was considered as the second cause of HL in Iranians and others (12). Moreover, TRIO and F-actin-binding protein (TRIOBP) gene are mapped on 22q13.1encoding TRIO and filamentous actin-binding protein with various isoforms is associated with profound ARNSHL (10, 11). DFNB93 is mapped, as a novel locus for ARNSHL, on 11q12.3–11q13.3 (16). Calcium Binding Protein 2 (CABP2) gene is a member of a subfamily of Ca^2+^ binding proteins that play an important role in the mammalian auditory system. CABP2 mutations in affected individuals who are from the DFNB93 affected family, lead to moderate-to-severe hearing loss in Iranian families (9).

Heterogeneous population of Iran with high consanguineous marriage rate (38.6%) and ethnic diversity offers a precious opportunity for genetic linkage analysis of ARNSHL (17). Hence, the present study was undertaken, for the first time, to analyze GJB2 and GJB3 genes mutations and to determine the contribution of three DFNB loci including DFNB4, DFNB28 and DFNB93 in ARNSHL families in Southern Iran.

## Materials and Methods

### Study subjects

Thirty-six large ARNSHL pedigrees with at least two affected subjects were included in this descriptive study from Hormozgan Province of Iran, during 2014 – 2015. Out of 36 families (167 individuals), 72 individuals had ARNSHL (43.11%) and 20 out of 36 studied families had consanguineous marriage (55.55%). HL screening for members of the families was performed by pure tone audiometric test and clinical examination, with special emphasis on recognizing potential environmental causes of hearing impairment such as infections, trauma, and information on exposure to known or possible ototoxic drugs or evidence of syndromic forms of deafness. The subjects divided into moderate (41–60 dB), severe (61–80 dB) and profound (≥80 dB) hearing impairment according to WHO classification of hearing loss. The frequencies of the moderate, severe and profound hearing loss among affected subjects were 11.11% (n=8), 58.33% (n=42), and 30.55% (n=22) respectively. HL informational questionnaires were obtained from all members of families and the pedigrees were drawn based on the filled-out questionnaires and interview with families by genetic counselors.

Ethics and Human Rights Committee of Hormozgan University of Medical Sciences approved the present study and written informed consent in accordance with the Declaration of Helsinki was obtained from all subjects for the purpose of study was explained.

### DNA extraction and GJB2 gene mutation screening

Genomic DNA was extracted from EDTA-containing peripheral blood of all members of the families using commercial kit (Genet Bio), according to the manufacturer’s instruction. All affected subjects were screened for mutations in exon 2 of the GJB2 gene using direct sequencing technique. To amplify the exon 2 of the GJB2 gene, the primers F: 5′- CGTCTTTTCCAGAGCAAACCG -3′ and R: 5′- AGCTCCATTGTGGCATCTGG -3′ were used in the PCR procedure. PCR thermal conditions were as follows: initial denaturation at 95 °C for 2 min, 35 cycles of denaturation at 94 °C for 30 sec, annealing at 59 °C for 30 sec, extension at 72 °C for 30 sec, final extension at 72 °C for 5 min. The amplified fragments of GJB2 gene (809 bp in length) were detected on 1% agarose gel. Subsequently, all amplified fragments were sequenced in both forward and reverse directions for confirmation of detected variations (Macrogen, Korea). We used CLC software to analyze the chromatograms and the sequencing results were compared with the Human Genome Database and GenBank.

### GJB3 gene mutation screening

Negative and heterozygous samples for GJB2 gene mutations were tested for GJB3. PCR was performed to amplify the entire coding sequence of GJB3 with F primer: 5′ GTCACCTATTCATTCATACGATGG3′ and R primer: 5′ TCACTCAGCCCCTGTAGGAC 3′. PCR thermal conditions were as follows: initial denaturation at 95 °C for 2 min, 35 cycles of denaturation at 94 °C for 30 sec, annealing at 60 °C for 30 sec, extension at 72 °C for 30 sec, final extension at 72 °C for 5 min. Finally, the PCR products (973 bp in length) were separated by electrophoresis procedure on 1% agarose gel. DNA sequencing of the PCR-amplified products was carried out bi-directionally (Macrogen, Korea), using the specific primers. Sequencing data were analyzed with CLC software and compared with the published sequences of Human Genome Database and GenBank.

### STR markers genotyping and linkage analysis

Negative samples for GJB2 gene mutations were tested for linkage analysis of DFNB4, DFNB28 and DFNB93 loci using STR markers that are shown in Table 1.

**Table 1:** STR markers of each locus and their primer sequences

***Locus***	***marker***	***Forward primer***	***Reverse primer***
DFNB4	D7S1817	CAAATTGGCAAAAACTGC	CCCCCCATTGAGGTTATTAC
	D7S523	CTGATTCATAGCAGCACTTG	AAAACATTTCCATTACCACTG
	D7S496	AACAACAGTCAACCCACAAT	GCTATAACCTCATAANAAACCAAAA
	D7S501	CACCGTTGTGATGGCAGAG	ATTTCTTACCAGGCAGACTGCT
DFNB28	D22S1045	GCTAGATTTTCCCCGATGAT	ATGTAAAGTGCTCTCAAGAGTGC
	D22S445	GTCCATCCGTTTGTTTGTTC	TGGATGGAGAGAAGGAATGA
	D22S272	GAGTTTTGTTTGCCTGGCAC	AATGCACGACCCACCTAAAG
	D22S1156	TGAGGTAGTCACACGAGGCA	AATTCACTGGGCTCCGAGG
DFNB93	D11S1765	CAGAAATGCCACCCAGAGAG	TTCCGGAGTTTGCACAATCT
	D11S1975	AGGACACAGCCTGCATCTAG	ACCAGGCATTGCACTAAAAG
	D11S1337	AAGGTGTGAGGATCACTGG	AGCTCATGGGGGCTATT

Based upon STRs physical distance in NCBI Map Viewer and NCBI UniSTS, we selected the STRs and their primers. A panel of 11 different STR markers was genotyped for DFNB4, DFNB28, and DFNB93 loci, according to the selection criteria including greater heterozygosity values, shorter amplicon and locating near the known locus. The PCR solutions contained 100 ng genomic DNA, 10×PCR buffer, 10 pmol of each primer, 10 nmol of each deoxyribonucleotide tri-phosphates, 1.5mmolMgCl2 and 1U Taq polymerase in a final volume of 25 μl. A two-step touchdown thermal cycling was designed for each STR markers based on the Tm of both primers. PCR was started with an initial denaturation step 94 °C for 5 min was followed by 7 cycles of denaturation (94 °C, 15 sec), annealing (60 °C, 40 sec) and extension annealing (72 °C, 40 sec) followed by a final 25 cycles of denaturation (96 °C, 40 sec), annealing (58 °C, 40 sec), and extension (72 °C, 40 sec) and final extension at 72 °C for 10 min. Finally, the PCR products were separated by electrophoresis procedure on 10% polyacrylamide gels stained with silver nitrate.

## Results

### Screening of GJB2and GJB3

**Fig. 1:**
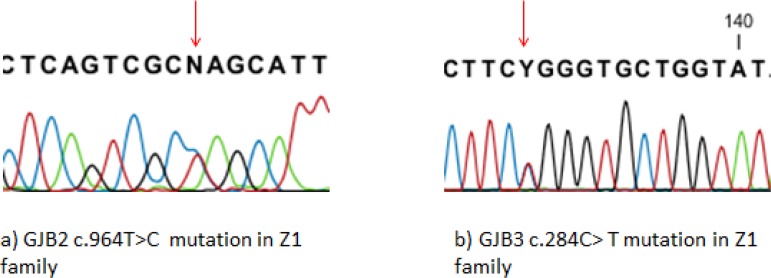
Results of GJB2 and GJB3 genes sequencing a) Chromatogram of GJB2 for Z1 family that shows c.964T>C mutation. b) Chromatogram of GJB3 for Z1 family which shows c.284C>T mutation

Altogether, four different heterozygous allelic variants were identified in the GJB2 and GJB3 genes in 8 of 36 (22.22%) studied families. DNA sequencing was identified a heterozygous allelic variant (964 C/T) (rs3751385) in 3′ untranslated region (3′ UTR) region of GJB2 gene in 5 out of 36 studied families (13.88%), while no common homozygous GJB2 mutations were detected. We found heterozygous variants in coding region of the GJB3 gene in three families (11.11%). Altogether, three different heterozygous mutations including 788G/A (rs61734064) (one family), 284C/T (rs1805063) (one family) and 973G/C (rs771511736) (one family) were identified. The 284C/T mutation in the GJB3 occur in compound heterozygosity along with the 964 C/T mutation in the GJB2 in one family (Fig. 1).

This family had two patients that two variants (GJB3 284C/T and GJB2 964 C/T) were detected in both of them.

### Linkage analysis of DFNB4, DFNB28 and DFNB93 loci

**Fig. 2:**
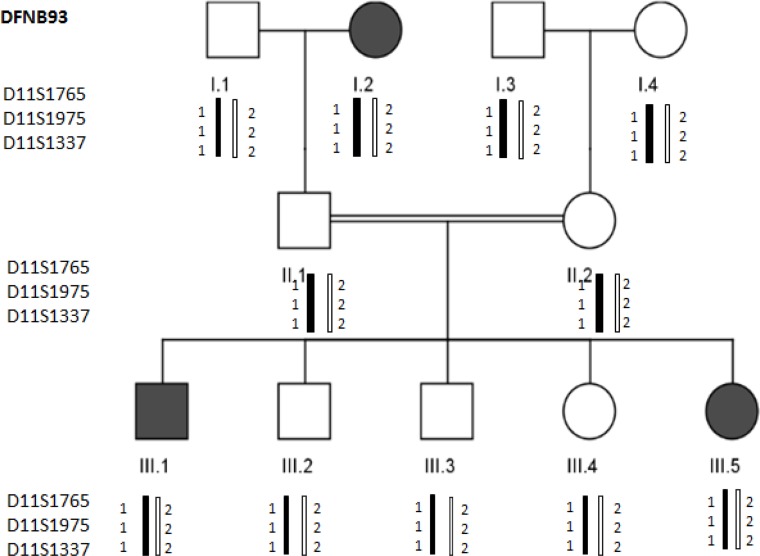
Pedigree of AH family and genotyping of STR markers (locus DFNB93)

We failed to identify linkage of the DFNB4, DFNB28 and DFNB93 loci among thirty-six GJB2 negative families. If at least three STR markers (fully informative) of a DFNB locus did not show homozygosity among the affected individuals of a family, then the locus would be determined unlinked. All members of GJB2 negative families, including parents, deaf and healthy siblings were heterozygous for all studied STR markers (Fig. 2).

## Discussion

DFNB loci including DFNB1, DFNB4, DFNB28 and DFNB93 is implicated in pathogenesis of ARNSHL in various populations (9–12). However, the role of these loci in pathogenesis of ARNSHL in south of Iran has not been investigated in depth. Therefore, this study aimed at analysis GJB2 and GJB3 gene mutations and to assess the correlation of the DFNB4, DFNB28 and DFNB93 loci in ARNSHL subjects.

We identified four different heterozygous allelic variants (GJB2 964 C/T, and 284C/T, 788G/A, and 973G/C variations in GJB3 gene) in 8 of 36 families. In three families (11.11%), we found heterozygous variants in coding region of the GJB3 gene. The 284C/T mutation in the GJB3 occur in compound heterozygosity along with the 964 C/T mutation in the GJB2 in one family. Analysis of the coding sequence of the GJB2 gene has failed to identify homozygous mutations required to cause the disorders in the families. The contribution of GJB2 gene in HL in different ethnicities of Iran is highly diverse and ranges from 27%–38% in a population of north to 0%–4% in the south-east of Iran (6, 7, 18). This discrepancy could be due to heterogeneity of ethnicity between various provinces of Iran.

Our results revealed heterozygous allelic variant (964C/T) in the GJB2 gene in 13.88% of studied families. These findings are in agreement with other studies which reported that 10%–50% of patients with ARNSHL carry a single heterozygous recessive mutation in the *GJB2* gene (17). Moreover, we found heterozygous variants including 788G/A, 284C/T and 973G/C in coding region of the GJB3 gene in 11.11% of the families.

In the last years, more than ten different *GJB3* mutations have been reported in deaf patients from various population such as Spanish, Brazilian, Turkish and Chinese (19–22). However, the pathogenicity of some of these variations remains disputed. Our results showed that 284C/T mutation in the GJB3 occur in compound heterozygosity along with the 964C/T mutation in the GJB2 in one family. This is in harmony with previous publications having reported different *GJB3* mutations occurred in compound heterozygosity with the *GJB2*gene in unrelated families(23). We advance the hypothesis that a genetic interaction between GJB2 and GJB3 genes could be lead to ARNSHL.

We unveil any linkage of the DFNB4, DFNB93 and DFNB28 loci among GJB2 negative families. Contrasting results have reported that 8%–15% of Iranian families with ARNSHL were linked to DFNB4 locus (3, 24). Mutations in the TRIOBP gene (DFNB28) were identified as a cause of prelingual, profound ARNSHL in a Palestinian, Pakistani and Indian families (25).

Mutations in a Novel Isoform of TRIOBP That Encodes a Filamentous-Actin Binding Protein Are Responsible for DFNB28 Recessive Nonsyndromic Hearing Loss--- Mutations in a novel isoform of TRIOBP that encodes a filamentousactin binding protein is responsible for DFNB28 recessive nonsyndromic hearing loss.

In accordance with our findings, no linkage was found to DFNB28 locus among 36 Iranian families with ARNSHL(3). In contrast, in another study, 5 out of 144 families were linked to this locus in Iranian subjects (26). DFNB93 locus linked to ARNSHL in 3 out of 37 of Iranian families for the first time (9, 16). We failed to find linkage of the DFNB93 locus among studied families.

## Conclusion

13.88% and 11.11% of studied families carried a heterozygous mutation for GJB2 andGJB3 gene, respectively. Moreover, it stresses the possibility that heterozygous mutations in GJB2 and GJB3 genes can be associated with ARNSHL. We did unveil any linkage of the DFNB4, DFNB28 and DFNB93 loci among GJB2 negative families. This disagreement between the reports could be attributed to racial differences populations or using a low number of subjects in the present study. Therefore, further studies on large-scale population will be needed to conclusively find linkage of DFNB4, DFNB28 and DFNB93 loci, and ARNSHL.

## Ethical considerations

Ethical issues (Including plagiarism, informed consent, misconduct, data fabrication and/or falsification, double publication and/or submission, redundancy, etc.) have been completely observed by the authors.
